# High molecular weight adiponectin levels are inversely associated with adiposity in pediatric brain tumor survivors

**DOI:** 10.1038/s41598-020-75638-w

**Published:** 2020-10-29

**Authors:** Rebecca Ronsley, Shahrad Rod Rassekh, Adam Fleming, Brianna Empringham, William Jennings, Carol Portwine, Sarah Burrow, Shayna Zelcer, Donna L. Johnston, Lehana Thabane, M. Constantine Samaan

**Affiliations:** 1 Division of Hematology, Oncology and BMT, Department of Pediatrics, University of British Columbia, Vancouver, BC Canada; 2 Department of Pediatrics, McMaster University, Hamilton, ON Canada; 3 Division of Pediatric Hematology/Oncology, McMaster Children’s Hospital, Hamilton, ON Canada; 4 Division of Pediatric Endocrinology, McMaster Children’s Hospital, Hamilton, ON Canada; 5 Division of Pediatric Orthopedics, Department of Surgery, McMaster University, Hamilton, ON Canada; 6 Division of Pediatric Hematology Oncology, Children’s Hospital, London Health Sciences Center, London, ON Canada; 7 Division of Pediatric Hematology/Oncology, Children’s Hospital of Eastern Ontario, Ottawa, ON Canada; 8 Department of Health Research Methods, Evidence and Impact, McMaster University, Hamilton, ON Canada; 9 Department of Anesthesia, McMaster University, Hamilton, ON Canada; 10 Centre for Evaluation of Medicines, St. Joseph’s Health Care, Hamilton, ON Canada; 11 Biostatistics Unit, St Joseph’s Healthcare-Hamilton, Hamilton, ON Canada; 12 Michael G. De Groote School of Medicine, McMaster University, Hamilton, ON Canada

**Keywords:** Endocrine system and metabolic diseases, Cancer, CNS cancer, Paediatric cancer, Biomarkers, Endocrinology, Oncology

## Abstract

While children with brain tumors are surviving at record rates, survivors are at risk of cardiovascular disease and type 2 diabetes mellitus; these conditions may be driven by excess body fat. Adiponectin in an adipokine that is inversely associated with the fat mass, and has been linked to cardiometabolic risk stratification in the general population. However, adiponectin’s profile and determinants in SCBT have not been established. We tested the hypothesis that high molecular weight (HMW) adiponectin levels, the more biologically active form of adiponectin, were associated with adiposity in SCBT similarly to non-cancer controls. Seventy-four SCBT (n = 32 female) and 126 controls (n = 59 female) who were 5–17 years old were included. Partial correlations and multivariable regression analyses assessed the relationship between HMW adiponectin and adiposity. HMW adiponectin was inversely associated with total and central adiposity (FM%: β − 0.21, 95% CI − 0.15, − 0.08; *p* value < 0.0001; WHR: β − 0.14, 95% CI − 0.02, − 0.01; *p* value < 0.0001 ;WHtR: β − 0.21, 95% CI − 0.05, − 0.03; *p* value < 0.0001). In conclusion, HMW adiponectin is inversely correlated with adiposity in SCBT. Adiponectin may serve as a biomarker of cardiometabolic risk and response to interventions to prevent and manage obesity and its comorbidities in SCBT.

## Introduction

One-third of the world’s population is overweight or obese, and this figure includes tens of millions of children^1–5^. These rates are significant, as childhood obesity persists into adulthood, and obesity-driven cardiovascular diseases and type 2 diabetes mellitus contribute to 4,000,000 adult deaths globally^6,7^. Obesity is a driver of the global epidemic of chronic non-communicable diseases, and is a significant public health challenge^6^.

Survivors of childhood brain tumors (SCBT) is an emerging group of childhood cancer survivors; this population exhibits vulnerability to premature cardiovascular disease and type 2 diabetes mellitus and early mortality when compared to non-cancer control groups^8–10^. There are multiple reasons for the susceptibility to cardiometabolic disorders in survivors, and an urgent need for interventions that can lower their risk to improve life expectancy and quality of life.

Obesity in children is characterized by the disproportionate expansion of the adipose tissue when compared to muscle and bone compartments^11–13^, with multiple biological, psychological, and lifestyle drivers of the adipose phenotype^14,15^. Importantly, while SCBT have excess adiposity when compared to controls, the potenial alteration in adipose tissue function is less well understood and may impact metabolic health^16^.

One of the most abundant products of the adipose tissue is the adipokine adiponectin. This adipokine has low, medium, and high molecular weight (HMW) isoforms^17^. The latter isoform is composed of 12–18 monomers and is considered the more relevant active form through which adiponectin exerts its biological actions^18^.

In the general population, obesity is associated with lower circulating adiponectin levels, which are linked to insulin resistance and independently predicts cardiovascular risk^19–22^. Also, weight loss improves circulating adiponectin levels and insulin sensitivity^23^. While studies have described HMW adiponectin profile in children, there are no studies on the association of HMW adiponectin with adiposity in SCBT^24–26^.

We tested the hypothesis that HMW adiponectin was associated with adiposity in SCBT. We also hypothesized that the HMW adiponectin profile was similar in SCBT and non-cancer controls.

## Results

Table 1 reports on baseline demographic, anthropometric, and clinical characteristics of the study participants. The study enrolled 74 SCBT and 126 non-cancer controls.

**Table 1:** Baseline characteristics of participants.

Variables	SCBT	Controls
	(Mean ± SD, n = 74)	(Mean ± SD, n = 126)
Age (years)	15.10 ± 7.30 (range 5.20–42.70)	14.00 ± 2.70 (range 5.40–18.80)
Height (cm)	151.10 ± 25.20	162.20 ± 15.10
Weight (kg)	53.30 ± 24.60	60.10 ± 22.00
BMI z-score	0.45 ± 1.20	0.52 ± 1.10
Body fat percentage (n = 180)	25.00 ± 10.00 (range 9–51)	22.70 ± 9.80 (range 6–47)
Waist-to-hip ratio (n = 198)	0.87 ± 0.08 (range 0.71–1.06)	0.83 ± 0.10 (range 0.65–1.28)
Waist-to-height ratio (n = 198)	0.48 ± 0.07 (range 0.36–0.75)	0.45 ± 0.08 (range 0.33–0.79)
Systolic blood pressure (mmHg)	105.00 ± 12.00	108.00 ± 11.00
Diastolic blood pressure (mmHg)	66.00 ± 9.00	68.00 ± 9.00
Adiponectin (µg/mL, n = 49 SCBT, n = 101 controls)	6.40 ± 5.40	18.70 ± 3.90

*BMI* body mass index, *cm* centimeter, *kg* kilogram, *SCBT* survivors of childhood brain tumors, *SD* standard deviation.

In SCBT group, the brain tumor types included non-Neurofibromatosis-1 (NF1) (N = 31, 41.90%) and NF1-related low-grade glioma (N = 11, 14.90%), medulloblastoma (N = 16, 21.60%), germ cell tumors (N = 6, 8.10%), subependymal giant cell astrocytoma (N = 3, 4.10%), ependymoma (N = 2, 2.70%), craniopharyngioma (N = 2, 2.70%), meningioma (N = 1, 1.40%), and other (N = 2, 2.70%). Tumors were distributed almost equally between the supratentorial (n = 35, 47.30%) and infratentorial (n = 39, 52.70%) compartments. The majority of SCBT were treated with surgery (n = 57, 77.00%), with 30 (40.50%) receiving radiotherapy and 36 (48.60%) receiving chemotherapy.

Both SCBT and non-cancer controls had similar age (SCBT: 15.10 ± 7.30 years; controls 14.00 ± 2.70 years) and sex distributions (SCBT: n = 32 female, 43.20%; controls: n = 59 female, 46.80%). Of note, the majority of SCBT were pubertal (n = 109 (86.50%); male n = 59 (88.10%); female n = 50 (84.70%)), and a similar trend was noted in controls (n = 51 (68.90%); male n = 30 (71.40%); female n = 21 (65.60%).

The mean BMI percentile was 64.20% for females and 61.40% for males, while body fat percentage was 26.70% in females compared to 20.10% in males.

One-in-four SCBT and one-in-three controls were overweight or obese based on BMI z-score measures (SCBT: overweight n = 13 (18.00%), obesity n = 7 (9.00%); controls: overweight n = 22 (17%), obesity n = 23 (18.00%)).

The levels of adiponectin were similar in SCBT and controls (6.40 ± 5.40 versus 18.70 ± 3.90 µg/mL, *p* value 0.589). Females had a higher level of HMW adiponectin when compared to male participants (6.90 ± 4.80 versus 4.80 ± 3.80 µg/mL, *p* value 0.041).

To determine the correlations between the fat depots and HMW adiponectin in SCBT and controls, we conducted partial correlation analyses adjusting for age, sex, and puberty (Table 2). Total adiposity (fat mass percentage, FM%) correlated with central adiposity measures (waist-to-hip ratio (WHR), waist-to-height ratio (WHtR)). Adiponectin was negatively correlated with FM% and WHtR in survivors but not in controls; there was no correlation between adiponectin and WHR in both populations (Table 2).

**Table 2:** Partial correlations of the different fat depots and adiponectin adjusted for age, sex, and puberty in SCBT and controls.

Population	Variables	Correlations (*p* value)	FM%	WHR	WHtR	Adiponectin
**SCBT**	FM%	r	1	0.4	0.77	− 0.42
		*p* value	–	0.006	< 0.0001	0.015
	WHR	r	0.4	1	0.61	− 0.2
		*p* value	0.006	–	< 0.0001	0.27
	WHtR	r	0.77	0.61	1	− 0.37
		*p* value	< 0.0001	< 0.0001	–	0.032
**Controls**	FM%	r	1	0.3	0.77	− 0.14
		*p* value	–	0.001	< 0.0001	0.161
	WHR	r	0.3	1	0.68	− 0.12
		*p* value	0.001	–	< 0.0001	0.27
	WHtR	r	0.77	0.68	1	− 0.14
		*p* value	< 0.0001	< 0.0001	–	0.171

*FM%* fat mass percentage, *95% CI* 95% confidence interval, *SCBT* survivors of childhood brain tumors, *WHR* waist-to-hip ratio, *WHtR* waist-to-height ratio.

To determine the relationship between HMW adiponectin and adiposity, we performed a multivariable linear regression analysis and adjusted for age, sex, puberty, cancer diagnosis, and treatments, including surgery, radiotherapy, and chemotherapy (Table 3).

**Table 3:** Regression analysis for the association of adiponectin, age, sex, and cancer status in SCBT and controls.

Variables	β	95% CI		*p* value
		Upper	Lower	
**Dependent variable: FM%**				
Adiponectin	− 0.21	− 0.15	− 0.08	< 0.0001
Age	0.26	0.23	0.53	< 0.0001
Sex	0.41	0.13	0.18	< 0.0001
Cancer status	0.16	0.04	0.1	< 0.0001
**Dependent variable: waist-to-hip ratio**				
Adiponectin	− 0.14	− 0.02	− 0.01	< 0.0001
Age	0.12	0.01	0.07	0.016
Sex	− 0.13	− 0.02	− 0.01	< 0.0001
Cancer status	0.19	0.012	0.024	< 0.0001
**Dependent variable: waist-to-height ratio**				
Adiponectin	− 0.21	− 0.05	− 0.03	< 0.0001
Age	0.27	0.08	0.18	< 0.0001
Sex	− 0.004	− 0.01	0.01	0.91
Cancer status	0.11	0.01	0.03	0.001

*95% CI* 95% confidence interval, *SCBT* survivors of childhood brain tumors, *WHR* waist-to-hip ratio, *WHtR* waist-to-height ratio.

The HMW adiponectin was negatively associated with total and central adiposity measures (FM%: β − 0.21, 95% CI − 0.15, − 0.08, *p* value < 0.0001; WHR: β − 0.14, 95% CI − 0.02, − 0.01, *p* value < 0.0001; WHtR: β − 0.21, 95% CI − 0.05, − 0.03, *p* value < 0.0001). Having a brain tumor diagnosis and older age were associated with adiposity, as well as female sex except for WHtR. Treatments and puberty had no significant association with adiposity.

In conclusion, HMW adiponectin was inversely associated with total and central adiposity measures in SCBT and non-cancer controls.

## Discussion

Over the past few decades, the number of children surviving brain tumors has reached record rates. However, survivors are at risk of developing obesity, cardiovascular disease, and type 2 diabetes mellitus^9,10,27–32^. While childhood obesity and dysglycemia drive early adult mortality^33^, SCBT have higher rates of adverse cardiometabolic outcomes and premature mortality than the general population but with similar BMI-defined obesity rates^9, 10,16,27–32,34^.

Importantly, SCBT have higher adiposity at similar BMI levels when compared to non-cancer controls^16,35^, and while the fat mass is a component of total body mass, it is a superior predictor of cardiometabolic risk in the general population versus BMI^36–43^.

Therefore, identifying biomarkers of the fat mass may help clarify which SCBT are at risk of cardiometabolic morbidities and mortality to target them with interventions that attempt to improve outcomes. This study has demonstrated that circulating HMW adiponectin is a biomarker of the fat mass in SCBT and that it follows the same trends observed in the non-cancer control group.

Adiponectin is a 30 kDa molecule secreted predominantly by adipocytes.

The endoplasmic reticulum plays an important role in regulating the synthesis and secretion of adiponectin, whereby Endoplasmic Reticulum resident protein 44 (ERp44) inhibits adiponectin secretion by retaining its oligomers^44^. The Endoplasmic Reticulum Oxidoreductase 1 alpha (Ero1-La) releases the ERp44-bound oligomers^45^. Adiponectin has significant Immunometabolic effects, including actions in insulin sensitivity and inflammation^19,46^. The mechanisms of adiponectin downregulation in obesity are not fully understood, but it may be related to adipose tissue macrophage secretion of Tumor Necrosis Factor Alpha (TNFα) and oxidative stress suppressing adiponectin gene expression^47–51^.

The HMW adiponectin levels trended higher in females when compared to male participants. Our results are consistent with current data in the general pediatric population^26,46,52–55^, with obese boys having the lowest levels when compared to girls and lean children, as the androgen rise with puberty in boys is associated with reduced adiponectin level^55,56^. In our cohort, the majority of participants were pubertal, and the number of prepubertal subjects was quite small and we could not verify the impact of puberty on adiponectin levels in survivors.

Children with central adiposity have lower adiponectin levels compared to those with normal central fat^52^. Also, patients with type 2 diabetes mellitus have lower adiponectin levels when compared to people with no diabetes^57^. This negative association of adiposity with adiponectin is consistent with the inflammatory response within visceral fat and immune cell infiltration and inflammatory cytokine secretion within this depot which may suppress adiponectin production^58^.

While SCBT have not had their adiponectin levels measured previously, pediatric leukemia and lymphoma patients have had their adiponectin profile interrogated. These studies demonstrated that adiponectin correlated with BMI-z score, sex, and puberty^59–62^. Our data highlight that the adipose tissue of brain tumor survivors likely has similar biological profile to that of survivors of other pediatric cancers and the general pediatric population, and further study of the adipose tissue is needed to understand the impact of the tumor and its treatment on adipose tissue Immunometabolism.

The role of adiponectin as an anti-inflammatory, and at times as an inflammatory, molecule has been debated^17,63^. Further analysis of adiponectin actions in SCBT is needed as a biomarker of adipose mass and to assess responses to interventions targeting adiposity and cardiometabolic risk.

One strength of this analysis involved the use of measures of total and central adiposity in assessing the relationship between adiponectin and the adipose profile in SCBT. Furthermore, we were able to compare SCBT to a healthy non-cancer control group in our analysis, providing a unique comparison population for this study.

One limitation of this study is that due to its cross-sectional design, it was not possible to elicit longitudinal changes in HMW adiponectin levels from diagnosis and into follow-up, and link that profile with future health outcomes. Furthermore, as the sample size is relatively small, tumor subgroup analyses were not possible to maintain the power of the study.

Future studies need to assess survivors with different tumors at sufficient numbers longitudinally to determine the potential association of adiponectin with other metabolic parameters in this population including glucose homeostasis. Whether adiponectin during childhood can predict future cardiometabolic outcomes in SCBT requires further assessment.

## Conclusions

In this study, adiposity in SCBT was inversely associated with HMW adiponectin levels. This population is highly vulnerable to adverse cardiometabolic disorders.

The insulin-sensitizing, anti-inflammatory, and anti-atherogenic effects of adiponectin makes it a critically vital molecule to study in survivors, as it may serve as a biomarker of future cardiovascular and type 2 diabetes risk. Adiponectin may also act as a marker of response to interventions aiming to prevent, delay, and treat obesity and its cardiometabolic comorbidities in pediatric brain tumor survivors.

## Methods

### Study design

This study was a cross-sectional investigation conducting a secondary analysis of data from the Canadian Study of Determinants of Endometabolic Health in ChIlDrEn (CanDECIDE study) cohort. The full protocol for this study and its feasibility has been previously published^64,65^. This study was approved by the Hamilton Integrated Research Ethics Board, and the methods adhered to the relevant regulations and guidelines.

### Participants

Participants were recruited from the endocrine, oncology, and orthopedics clinics at McMaster Children’s Hospital, a Tertiary Pediatric Academic Center in Hamilton, Ontario, Canada. Participants included children between 5–17 years of age, who are either lean, overweight, or obese based on their body mass index z-score (BMI z-score) as defined by standard criteria^66^. SCBT had to have completed therapy for at least six months before study enrolment.

Participants were excluded due to active infections or a history of infections within 14 days before participation, history of autoimmune disorders, or using immunosuppressive therapy or systemic steroids at a dose higher than maintenance dosing (6–8 mg/m^2^/day), or had a history of smoking. We excluded participants and families who were unable to provide consent^64,65^.

Participants 16 years and older provided written informed consent. For participants between 7 and 15 years of age, the parent or guardian supplied written informed consent, and the participant provided assents. For those below seven years of age, the parent or guardian provided written informed consent before study inclusion. The study participation rate was 26.8% as previously reported^65^.

### Data collection

We collected clinical measures, including height, weight, waist circumference, hip circumference, blood pressure, and adiposity data. Questionnaires were also administered to participants’ parents and to participants at study visits to collect sociodemographic data, past medical history, and pubertal stage (using validated Tanner pubertal staging pictures)*.* Clinical data, including diagnosis and treatment details, were also collected and verified from medical notes.

### Clinical measures and variable definitions

We collected anthropometric measures including height that was assessed with a stadiometer and measured to the closest 0.1 cm. We measured weight to the nearest 0.1 kg using an electronic scale (Seca, USA). We calculated the BMI using a standard formula of weight (kg)/[height (m)]^2^ and the BMI percentile using the Children’s BMI Tool for Schools^66^. We measured the BMI z-score using the Centers for Disease Control and Prevention growth chart^67^. We measured Total adiposity by bioelectrical impedance analysis using the Tanita body fat monitor (Tanita Corporation, Illinois, USA). We measured waist circumference and hip circumference using a spring-loaded tape measure. We then calculated the waist-to-hip and waist-to-height ratios to assess central adiposity^64,65^.

### Enzyme-Linked Immunosorbent Assay (ELISA) for adiponectin

We collected blood samples into EDTA tubes in the fasted state. Centrifugation of the samples took place at room temperature for 15 min at 1,500 g to isolate plasma. We stored the samples in cryovials at − 80 °C. When preparing the samples for the assay, the samples were centrifuged at room temperature for 15 min at 1500 g after they were thawed on ice. The high molecular weight Adiponectin levels were quantified using the commercially available enzyme-linked immunosorbent assay (ELISA) Human HMW Adiponectin/Acrp30 Quantikine ELISA Kit (R&D Systems, Minneapolis, USA) as per manufacturer’s guidelines^68^.

### Statistical analysis

We used the method of Norman and Streiner to calculate sample size^69^. We report the demographic data and baseline variables using descriptive analyses based on the variable type. We report continuous variables as mean ± SD and categorical variables as numbers (%). We tested the data for normality of distribution using the Shapiro–Wilk test, and non-normally distributed data were log-transformed. We tested for colinearity using variance inflation factor.

We imputed missing data in SPSS and included five imputations per missing variable. After variable selection, the output dataset encompassed our original data set with the imputed missing data and a set of cases with imputed values generated by the program^70^. The variables that were imputed included fat mass percentage (n = 20/200), waist-to-hip ratio (2/200), waist-to-height ratio (2/200), and adiponectin (n = 50/200). The validity of our methods has already been established with the percentage of imputations that will maintain data validity^70–73^.

We used partial correlations to assess adiponectin’s association with fat mass percentage, WHR, and WHtR and adjusted for age, sex, and puberty. An independent sample t-test was used to assess the differnces in adipose depots and total and sex-specific differences in HMW adiponectin levels.

We conducted a multivariable regression analysis to assess the relationship between adiposity and HMW adiponectin. Adiposity measures used included total adiposity (FM%) and central adiposity using WHR and WHtR measures. These analyses had age, sex, puberty, cancer diagnosis, and treatments included as independent variables. Two subjects per variable were needed to address the association of adiponectin with adiposity^74^. Therefore, the number of participants in the SCBT and the control groups has provided valid results.

As fat mass percentage and BMI z-score were co-linear variables, we included only the FM% in the analysis. We report the results as standardized β coefficients with 95% CI and associated *p* values. SPSS version 25.0 was used to conduct all analyses^75^.

## Data Availability

The study data are available from the corresponding author upon reasonable justification.
